# Synthesis of Flower-like Nickel Hydroxide Nanosheets and Application in Electrochemical Determination of Famotidine

**DOI:** 10.22037/ijpr.2019.14257.12245

**Published:** 2020

**Authors:** Rezvan Dehdari Vais, Hossein Yadegari, Hossein Heli

**Affiliations:** a *Nanomedicine and Nanobiology Research Center, Shiraz University of Medical Sciences, Shiraz, Iran. *; b *Department of Materials, Faculty of Engineering, Imperial College London, United Kingdom.*

**Keywords:** Nickel, Pepcid, Nanoflower, Nanomaterial, Electrocatalysis, Electroanalysis

## Abstract

An electrochemical sensor was designed and fabricated for electrocatalytic oxidation and determination of famotidine in pharmaceutical forms. The electrochemical oxidation process and its kinetics were investigated using cyclic voltammetry, steady-state polarization measurements, and chronoamperometry techniques, and also the analytical measurements were performed by amperometry. Upon addition of the drug into the solution, cyclic voltammograms of the fabricated sensor exhibited an increased anodic peak current associated with a decrease in the corresponding cathodic current. These results suggested an electrocatalytic EC’ oxidation mechanism for famotidine on the oxyhydroxide species immobilized on the electrode surface. Accordingly, a mechanism involving generation of Ni^3+^ active sites and their subsequent consumption by the drug was proposed. Moreover, the corresponding rate law under the control of charge transfer was developed and kinetic parameters were derived. A sensitive and time-saving amperometric procedure was also developed for the analysis of famotidine with a detection limit of 5.91 mmol L^-1^. Using the developed amperometric procedure, famotidine was successfully analyzed in the presence of ibuprofen. The developed sensor in this study displayed enhanced sensitivity and selectivity, compared to some other reported methods.

## Introduction

Drug analysis is a continuously growing research field that has brought a revolution in health promotion. It plays a meaningful role on patient’s samples in order to support clinical testing, bioavailability and pharmacokinetic purposes, drug intoxication, and therapeutic drug monitoring (1). Therefore, it is essential to develop more sensitive and accurate methods for the quantitative determination of drug substances comprising active ingredients, pharmaceutical excipients in solid or liquid dosage forms, and their metabolites in biological fluids. For this purpose, electrochemical techniques are often preferred for the drug analysis because of their abilities to provide higher sensitivity and selectivity, lower detection limit, reduced sample size and solvent consumption, shorter analysis time and lesser operating cost, when compared to other conventional methods. In addition to the above-mentioned factors, electroanalysis has been extensively used to estimate adsorption-diffusion parameters, oxidation-reduction characteristics, and plausible mechanism pathways of redox-active drugs (2, 3).

Meanwhile, nanostructured materials are extremely attractive in a vast area of applications due to the unique properties arising from their high real surface area and small size. These nanomaterials can be synthesized using a variety of methods, leading to structures with different sizes and shapes (3-7). Special morphology, excellent electronic conductivity and high electro/catalytic activity of nanomaterials result in accelerated electron transfer as well as increment in sensitivity and selectivity of detection, when these materials are employed to fabricate biosensing devices (3, 6-9).

Nanostructured transition metal oxides/hydroxides are considered as one of the most important groups of materials that exhibit unique and highly tunable properties compared to their bulk counterparts owing to their multiple oxidation states, symmetry, density, acid-base surface properties, crystal-field stabilization, stoichiometry, and coordination numbers. Moreover, good chemical stability, compatibility with the electrolytes, wide range of band gap, high dielectric constants, ease of preparation, and more environmentally benignant are the factors that distinguish transition metal oxides/hydroxides from other nanomaterials. These unique properties have made transition metal oxides/hydroxides to have potential applications in energy producing and storage devices, electrochromism, electronic devices as well as gas-, bio-, chemical and electrochemical sensors (10-14). Nickel (hydr)oxide is an important and abundant material from this group that has recently attracted tremendous interests in a broad range of technological applications and fundamental studies due to its distinctive features of superparamagnetism, non-toxicity, high reactivity and conductivity, large specific surface area, short diffusion path, stable interface, and increased active sites. Also, nanostructured nickel (hydr)oxides represent excellent electrocatalytic activity toward small organic molecules, attributed to its redox reaction under anodic potentials in alkaline electrolytes (15-17). The latter unique property is well proven through extensive research findings on nickel-based electrodes and their applications for amperometric detection of carbohydrates, amines and amino acids (16-18), homogeneous CO_2_ reduction (19) and electrocatalytic oxidation of methanol related to fuel cell technology development (20). Previous studies demonstrated that the experimental conditions and the preparation techniques play a critical role in controlling the size, morphology and properties of nanostructured nickel (hydr)oxides, which can affect the performance of the nickel-based electrodes (16, 17).

Nickel (hydr)oxide nanostructures can be synthesized using a variety of methods including microwave-assisted liquid-phase growth (21), sol-gel processing (22), chemical or electrochemical deposition (23), hydrolysis-cathodic deposition (24), and hydrothermal as well as solvothermal synthesis (25). Compared to other methods, hydrothermal synthesis process is a simple, one-step, and straightforward route that has been most commonly used in fabricating inorganic nanomaterials like metals and metal oxides or hydroxides with high purity, uniform grain structure, controlled morphology, size, and orientation. It has been proven that hydrothermal growth of such nanostructures is generally dependent on the used precursors, growth temperature, addition of surfactants and mineralizers or other chemicals, substrate type, pretreatment, and solution pH (15, 18).

Fabrication and characterization of chemically modified electrode materials are among the most active research areas due to their wide range of potential applications from electrochemical technology, energy conversion and chemical analysis to information storage, electrochromism devices, and displays (20). Surface modification of the electrode materials is applied to enhance the electron transfer kinetics as well as electrocatalytic reactions involving redox species with facilitated electrode reaction(s) and/or reducing overpotential required for oxidation/reduction of the target compounds. Immobilization of the surface-active materials switching between various valence states under the influence of an external electric field is of particular interest in this regard. Applying nanostructured materials as electrode modifiers have attracted a great deal of attention in recent years because of supplying unique size- and shape-dependent properties arising from size and quantum confinement (3-7, 9, 13, 14 and 26). 

**Scheme 1:**
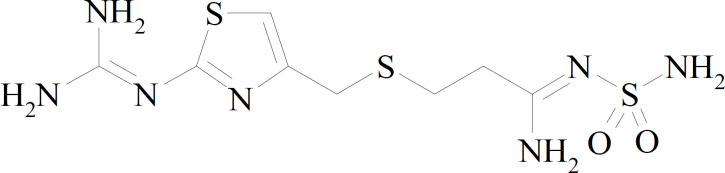
Chemical structure of famotidine.

Famotidine with chemical name of 3-[({2-[(diaminomethylidene)amino]-1,3-thiazol-4-yl}methyl)sulfanyl]-N-sulfamoylpropanimidamide blocks the histamine function of the stomach cells to inhibit acid production by the stomach (Scheme 1). The drug is administrated to treat duodenal ulcers and repress their recurrence, gastric ulcer, gastro esophageal reflux disease, and Zollinger-Ellison syndrome (27). Famotidine is also administered before operation to surgery patients to inhibit postoperative queasiness and reduction of aspiration pneumonitis risk and to prevent gastrointestinal toxicity (such as peptic ulcer) of nonsteroidal anti-inflammatory drugs such as ibuprofen.

The clinical advantages and extensive use of famotidine have directed the research activities towards developing efficient and reliable analytical techniques for the assay of famotidine in different media. Determination of famotidine in pharmaceutical forms and biological fluids have been carried out by means of spectrophotometry (28, 29), spectrofluorimetry (30), potentiometry (31), high performance liquid chromatography (HPLC) (32, 33), HPLC-mass spectrometry (34), gas chromatography (35), capillary zone electrophoresis (36), and electrochemistry (37).

Continuing our recent studies on the development of nanostructured materials to study the kinetics and mechanisms of electrooxidation/electroreduction of drugs (3, 7 and 9), in the present study we report the synthesis of flower-like nickel hydr-oxide nanosheets and its application in electrocatalytic oxidation and determination of famotidine via a mediated electron transfer process. To the best of our knowledge, electroanalysis of this drug using modified electrodes and mediated electron transfer kinetic has not been reported. The developed analysis method in this study can be served as a low-cost alternative technique for famotidine detection and measurement in pharmaceutical form and biological liquids.

## Experimental

All chemicals used were of analytical grade from Scharlau (Spain) or Merck (Germany) and were used as received. All solutions were prepared with doubly distilled water. Famotidine was received from Shafa Darou, Tehran, Iran. The famotidine tablets were purchased from a local drugstore. A standard solution of famotidine was prepared by dissolving the pure famotidine form in NaOH. The prepared standard solution was stored in dark at 4 ºC. Additional dilute solutions were prepared daily by accurate dilution just before use.

To synthesize flower-like nickel hydroxide nanosheets, 0.50 g Ni(NO_3_)_2_.6H_2_O and 0.15 g NaF were added into 75 mL ethanol and stirred to dissolve. The mixture was then transferred into a Teflon-lined stainless-steel autoclave and heated to 190 ºC for 50 h. The autoclave was then cooled down to room temperature naturally. The resultant green product was washed with a 1:1 water/ethanol mixture several times and dried at 50 °C.

Morphology of the synthesized nano-structure was evaluated with field emission scanning electron microscopy (FESEM) using a Zeiss, Sigma-IGMA/VP (Germany). Unmodified carbon paste electrode (UCPE) was prepared by careful hand-mixing of graphite powder and mineral oil with a ratio of 80/20% (w/w). The paste was packed firmly into a 3.0 mm diameter cavity at the end of a Teflon tube. Electrical contact was established by a copper wire. Flower-like nickel hydroxide nanosheets were employed as a modifier to prepare modified carbon paste electrodes (MCPEs) with different modifier ratios. Based on the results, the optimum modified electrode was obtained by mixing graphite fine powder, mineral oil, and the modifier at 74:20:6 ratios for about 10 min. To cover the MCPE surface with a Nafion layer to improve/provide selectivity of the electrode, 10 μL of a 2% w/v low aliphatic alcohols Nafion solution was dropped on the electrode surface and placed under an IR lamp to dry. Before applying MCPE, it was transferred to a 100 mmol L^-1^ NaOH solution and potential in the range of 0 to 700 mV were applied to the electrode at a potential sweep rate of 50 mV sec^-1^ for 50 cycles. This pretreatment procedure electrochemically stabilized the electrode surface.

Electrochemical studies were carried out in a conventional glass cell incorporating a three-electrode configuration containing 100 mmol L^-1^ NaOH as the running electrolyte, powered by a µ-Autolab electrochemical analyzer (Eco-chemie, The Netherlands). The system was run on a PC using GPES 4.9 software. An Ag/AgCl, 3 mol L^-1^ KCl and a glassy carbon rod were used as the reference and counter electrodes, respectively. Calibration curves were plotted by an amperometric technique using different concentrations of famotidine in 100 mmol L^-1^ NaOH solution. A working potential of 580 mV was applied for amperometric measurements in which the transient currents were allowed to decay to steady-state values.

For analysis of the drug tablets, an average mass of ten tablets was determined and then finely powdered and homogenized in a mortar. An accurately weighed amount of the homogenized powder was transferred into a 100 mL calibrated flask containing 50 mL of 100 mmol L^-1^ NaOH solution. The contents of the flask were sonicated for 30 min, then the undissolved excipients were removed by filtration and diluted to volume with the same supporting electrolyte. Appropriate solutions were prepared by taking suitable aliquots of the clear filtrate and diluting them with 100 mmol L^-1^ NaOH. All studies/measurements were carried out at room temperature.

## Results and Discussion

**Figure 1:**
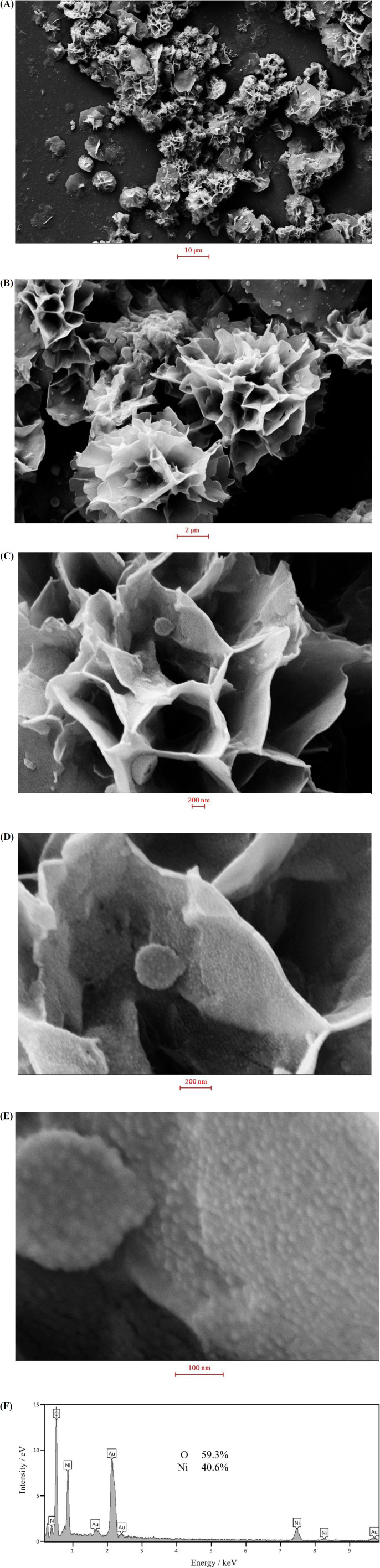
(A-E) SEM micrographs of the synthesized nickel hydroxide nanosheets at different magnifications; (F) EDS spectrum of the electrode surface

In order to investigate the morphology of the nanosheets, the synthesized nanostructures were examined by FESEM. FESEM images recorded at different magnifications and an EDS spectrum are shown in Figure 1. At low magnifications, the nickel hydroxide particles resemble to followers comprising interwoven sheets. The sheet thickness was estimated to be 35 ± 9 nm. At high magnifications, it can be seen that the sheet surfaces are covered by very small particles of 13 ± 3 nm. This special morphology provides holes and cavities which accelerate the mass transport of species, when the nickel hydroxide sample being in contact with an electrolyte. Meanwhile, the small nanoparticles present on the sheet surfaces increase the real surface area and hence the (re)activity of nickel hydroxide. EDS spectrum of the sample confirmed the purity and the chemical composition of the prepared sample to be Ni(OH)_2_. The formation of flower-like nickel hydroxide nanosheets can be explained by the wet-etching model (38). Fluoride ions act as coordination ligands, forming holes and cavities by fluoride-mediated coordination-assisted etching during the hydrothermal process of nickel ions (38).

**Figure 2:**
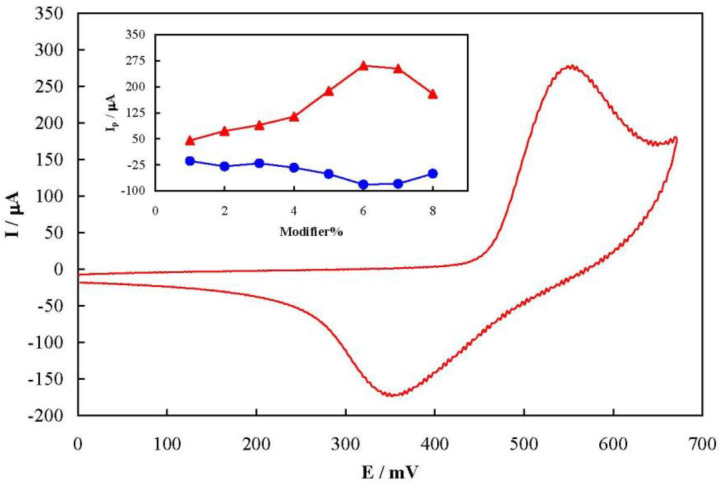
A typical cyclic voltammogram of MCPE prepared with 6% of the modifier recorded at a potential sweep rate of 50 mV sec^-^^1^ in 100 mmol L^-1^ NaOH solution. Inset: dependency of the anodic and cathodic peak currents on the percentage of the modifier in MCPE

Figure 2 shows a typical cyclic voltammogram of MCPE prepared with 6% of the modifier recorded at a potential sweep rate of 50 mV sec^-1^ in 100 mmol L^-1^ NaOH solution. The voltammogram pattern is similar to those reported previously in which the redox process is related to the solid state Ni^2+^/Ni^3+^ transition in an alkaline solution (16, 18). A pair of well-defined peaks with a mid-peak potential of 454 mV and a peak-to-peak potential separation of 200 mV appeared in the voltammogram. The peak-to-peak potential separation is higher than the theoretical value of zero indicating a quasi-reversible kinetics. The value of full width at half height of the anodic peak is estimated to be >140 mV. This is greater than the 90 mV, the theoretical value for non-interacting one-electron surface redox species (39). Therefore, there were repulsive forces between nickelaceous species immobilized on the MCPE surface (40).

The influence of the modifier amount on the MCPE voltammetry response is represented as dependency of the anodic and cathodic peak currents on the percentage of the modifier in MCPE in the inset of Figure 2. Based on the results, both anodic and cathodic peak currents increased upon increment of the amount of flower-like nickel hydroxide nanosheets up to 6% in the carbon paste, followed by a decrease due to the decrement in the electrical conductivity of the paste by further increase of the nickel hydroxide nanosheets. Therefore, 6% was selected as the optimum modifier amount in the fabrication of MCPE throughout the study.

**Figure 3:**
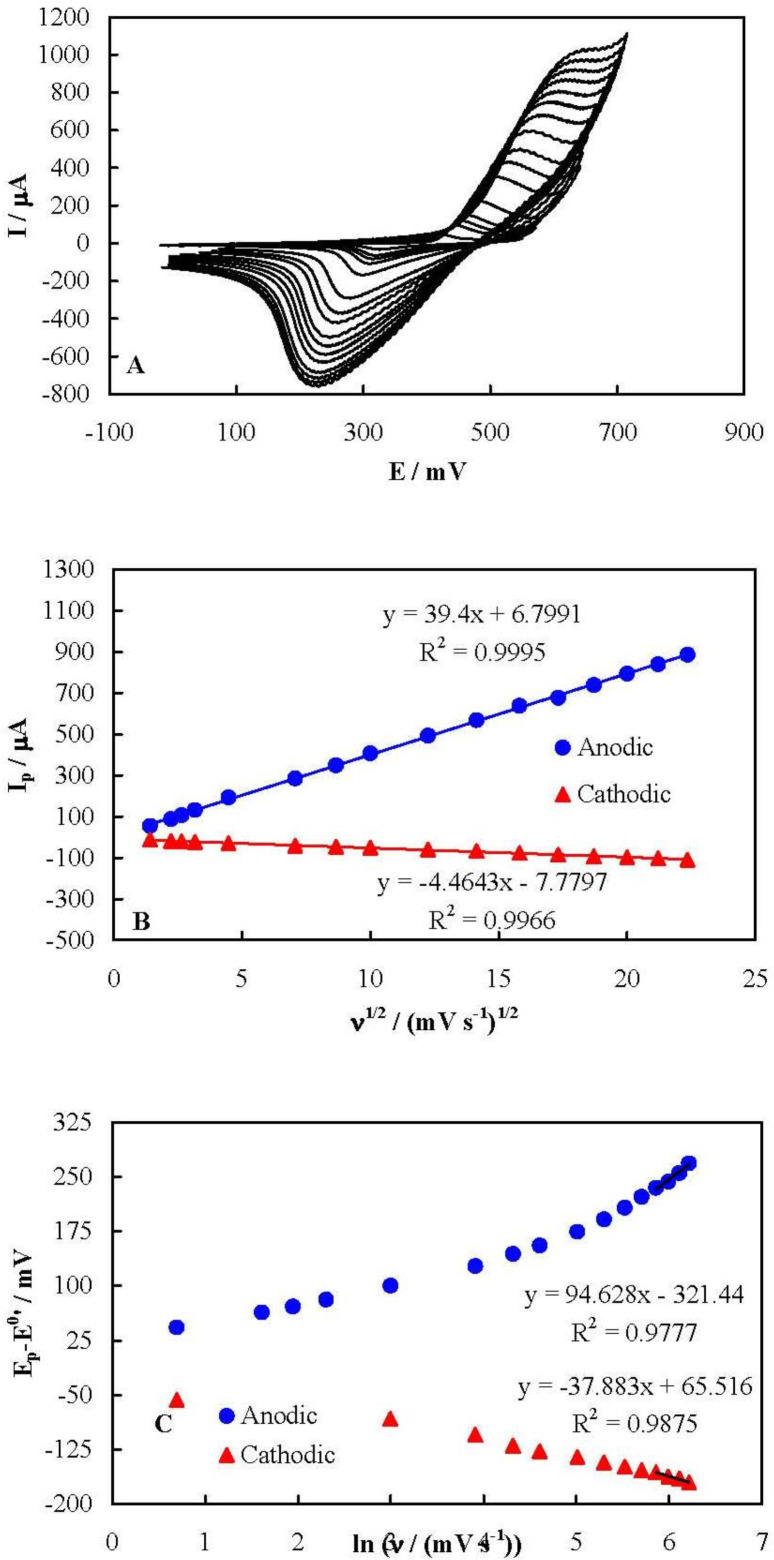
(A) cyclic voltammograms of MCPE recorded in 100 mmol L^-1^ NaOH solution at different potential sweep rates in a range of 2 to 500 mV sec^-1^, (B) dependency of anodic and cathodic peak currents on the corresponding square root of potential sweep rate; (C) dependency of anodic and cathodic peak currents on the natural logarithm of the potential sweep rate

Figure 3A represents cyclic voltammograms of MCPE recorded in 100 mmol L^-1^ NaOH solution at different potential sweep rates in a wide range of 2 to 500 mV sec^-1^. Upon increasing the potential sweep rate, the peak current increased and the peak potential shifted in an irreversible-kinetic manner for both anodic and cathodic peaks. It can be deduced from these voltammograms that both the anodic and cathodic peak currents depended linearly on the square root of the potential sweep rate in all of the tested potential sweep rates, as shown in Figure 3B. It should also be noted that the peak currents did not shown a linear dependency on the potential sweep rate, even at low potential sweep rate values, as is shown Figure S1 in Supplementary file. This behavior indicates that the kinetics of the redox process of the nickelaceous species on the surface of MCPE was purely controlled by diffusion without any limitation arising from the electron exchange resistance between the mediator and the underlying current collector. This implies that the flower-like nickel hydroxide nanosheets had a high electron transfer rate. It is noteworthy that there has been a limitation in the electron exchange between the nickelaceous species in some other nanostructures of nickel hydroxide, confirming from the dependency of the peak currents on the potential sweep rate at slow potential sweep rates (16-18). However, such a limitation was not observed here for the flower-like nickel hydroxide nanosheets.

The kinetic parameters of the nickel redox species can be obtained using the Laviron Equations (39). When the peak-to-peak potential separation is greater than 200/n mV (n is the number of exchanged electrons), these Equations are applicable:

E_pa_ = E^0^′ + X ln [(1-α_s_)/m]                    (Equation 1)

E_pc_ = E^0^′ + Y ln [α_s_/m]                      (Equation 2)

ln k_s_ = α_s_ ln (1-α_s_) + (1-α_s_) ln α_s_ - ln (RT/nFν) - α_s_ (1-α_s_) nF(E_p_-E^0^’)/RT                     (Equation 3)

X = RT/(1-/α_s_) nF                     (Equation 4)

Y = RT/α_s_nF                     (Equation 5)

m = (RT/F) (k_s_/nν)                      (Equation 6)

where E_p_ is the anodic or cathodic peak potentials, E^0^’ is the formal potential (equal to mid-peak potential of the voltammograms recorded at low values of ν), α_s_, k_s_,and ν are the electron transfer coefficient, apparent charge transfer rate constant and potential sweep rate, respectively. Based on these Equations, Figure 3C shows the plots of (Ep-E^0^') on ln (ν) for both the anodic and cathodic peak potentials. (E_p_-E^0^') showed a linear dependency on the natural logarithm of the potential sweep rate for the potential sweep rates greater than 350 mV sec^-1^. Using the slopes and intercepts of the plots and Equations 1 to 6, the values of α_s_ and k_s_ were obtained as 0.47 and 0.23 sec^-1^, respectively.

**Figure 4:**
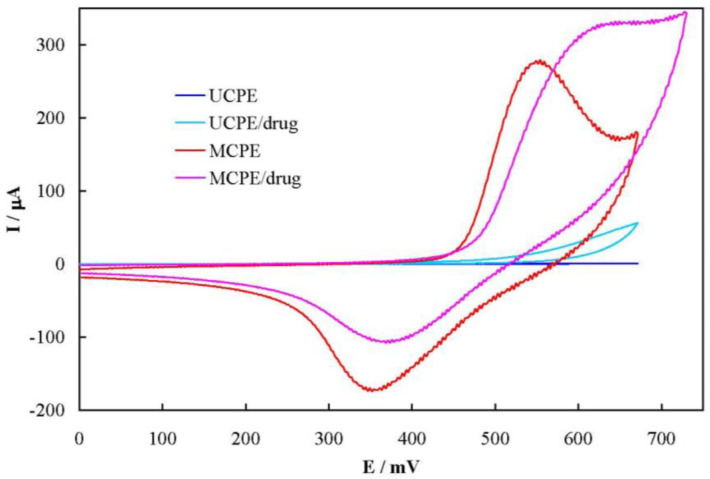
Cyclic voltammograms of UCPE and MCPE electrodes in 100 mmol L^-1^ NaOH solution in the absence and presence of 6.0 mmol L^-1^ famotidine, recorded at the potential sweep rate of 50 mV sec^-1^

Figure 4 shows cyclic voltammograms of UCPE and MCPE in 100 mmol L^-1^ NaOH solution in the absence and presence of 6.0 mmol L^-1^ famotidine, recorded at the potential sweep rate of 50 mV sec^-1^. For UCPE, a sluggish and ill-defined anodic peak current is appeared in the voltammogram in the presence of famotidine. Using MCPE; however, the anodic peak current and the related charge increased by 18 and 60% in the presence of the drug, respectively. In addition, MCPE shows a decrease in the corresponding cathodic current and the related charge by 37 and 63%, respectively. Based on these results, famotidine was oxidized by the higher valence nickel species (Ni^3+^), resulting in formation of lower valence one (Ni^2+^). Therefore, the apparent concentration of Ni^2+^ increased which in turn caucused increment in the anodic peak current and charge. Similarly, the reaction between the drug and Ni^3+^ continued in the backward sweep, resulting in the decrease of the Ni^3+^ concentration and decrement in the cathodic peak current and charge. This mediated oxidation (EC’ mechanism) of famotidine can be continued through the external electrical circuit by prolonged application of a positive potential. The cathodic current in the reverse sweep did not disappeared, indicating that the reaction between famotidine and Ni^3+^ was the rate-determining step in the overall oxidation process. Moreover, the onset potential of the anodic peak in the presence of the drug shifted toward more positive values, indicating the presence of a strong interaction between famotidine and Ni^3+^ at the MCPE surface.

**Figure 5:**
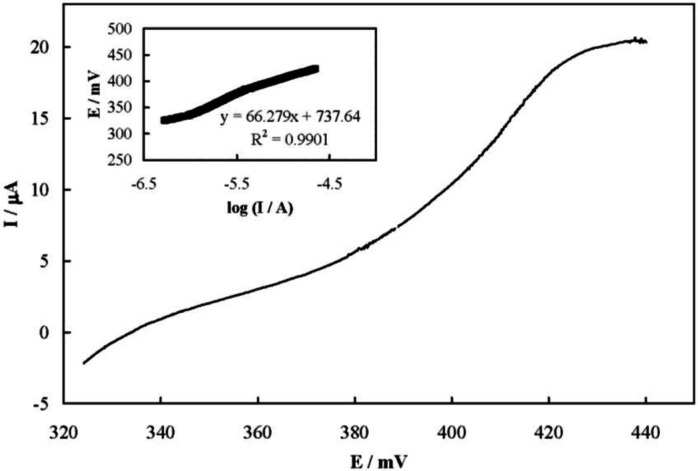
Steady-state current-potential (Tafel) plot recorded for the electrooxidation of famotidine on the MCPE surface

Figure 5 shows a steady-state current-potential plot recorded for the electrooxidation of famotidine on the MCPE surface which represents a S-shaped plot. The transfer coefficient of Ni^2+^ oxidation in the presence of famotidine (α) was obtained from the Tafel plot to be 0.4 (inset of Figure 5).

**Figure 6:**
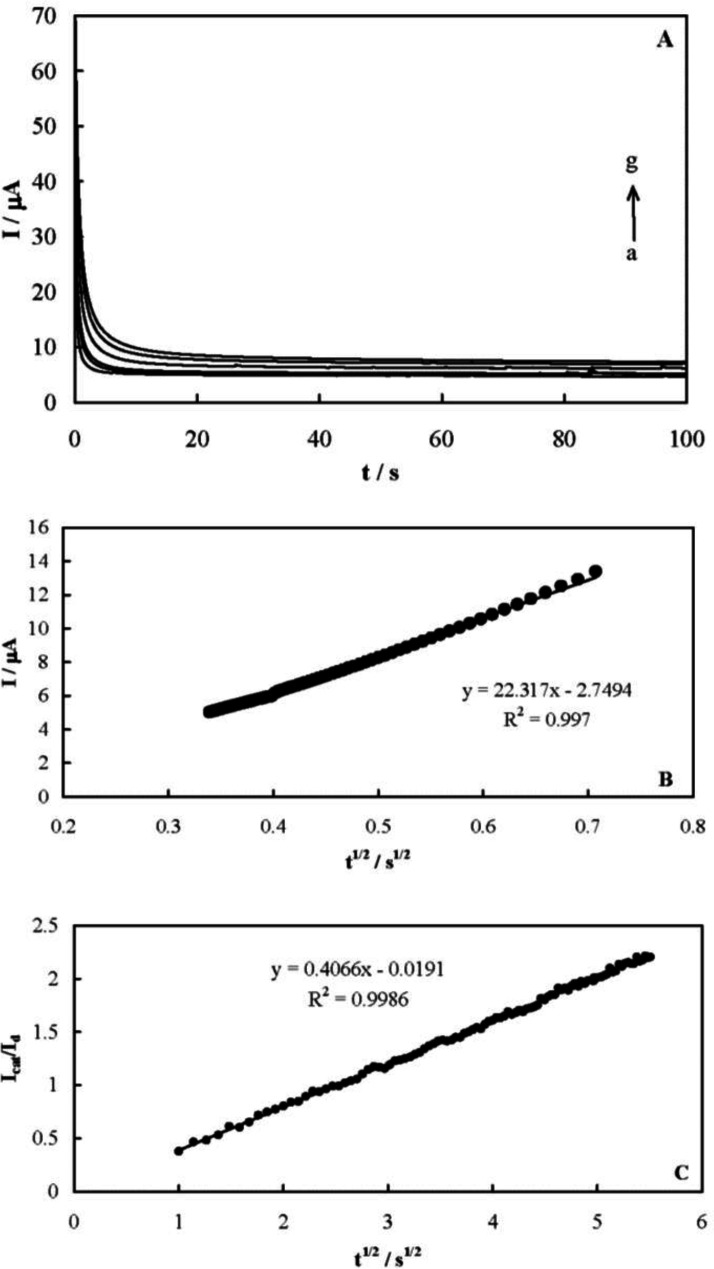
(A) The chronoamperometric response of MCPE recorded in the absence (curve a) and presence of famotidine (curves b-g) over a concentration range of 0.08-0.8 mmol L^-1^ with an applied potential step of 580 mV; (B) dependency of the net current versus the mines square root of time; (C) I_cat_/I_L_ *vs.* t^1/2^ plot for the recorded chronoamperogram

The chronoamperometric responses of MCPE were recorded to measure the catalytic rate constant of famotidine electrooxidation on the MCPE surface after setting the working electrode potential to a desired value. Figure 6A shows the obtained chronoamperograms in the absence and presence of famotidine over a concentration range of 0.08-0.8 mmol L^-1^. A potential step of 580 mV was applied during the measurements. The net currents with respect to the mines square root of time resulted in a linear dependency (Figure 6B), indicating a diffusion-controlled process for the electrooxidation of the drug. The coefficient of diffusion of famotidine can be obtained according to the Cottrells’ Equation and using the slop of this line (41):

I = nFAD^1/2^C^*^π^-1/2^ t^-1/2^                     (Equation 7)

where D is the coefficient of diffusion and C^*^ is the bulk concentration. A mean value for the diffusion coefficient of famotidine was measured to be 5.33 × 10^-6^ cm^2^ s^-1^.

The catalytic rate constant of the electrooxidation reaction can also be obtained using chronoamperometry according to (41):

I_cat_/I_L_ = γ^1/2^[π^1/2^erf (γ^1/2^) + exp (-γ)/γ^1/2^]                      (Equation 8)

where I_cat_ and I_L_ are the currents in the presence and absence of the drug, respectively, and γ = k’C^*^t is the argument of the error function. k’ is the catalytic rate constant and t is elapsed time. For γ > 1.5, erf(γ^1/2^) is almost equal to unity and the above Equation can be reduced to:

I_cat_/I_L_ = γ^1/2^ π^1/2^_=_ π^1/2^ (k’C^*^t)^1/2^                     (Equation 9)

Based on the slope of the I_cat_/I_L_ *vs.* t^1/2^ plot presented in Figure 6C, a mean value of k’ for the electrooxidation of famotidine was obtained as 6.6 × 10^4^ cm^3^ mol^-1^ s^-1^. The following reaction mechanism is proposed based on the presented results for the mediated oxidation of famotidine on the MCPE surface. Ni^2+^ oxidizes to Ni^3+^ in the first step:

Ni(II) Ni(III) + e^-^                      (Equation 10)

The next step involves the oxidation of famotidine on the modified surface via the following reaction:

Ni(III) + famotidine → Product + Ni(II)                      (Equation 11)

As for the product(s) of the famotidine electrooxidation, the drug possesses four primary amine functional groups, which are likely oxidized to the corresponding hydroxylamine (42):

R-NH_2_ + H_2_O→ R-NHOH + 2H^+^ + 2e^-^                     (Equation 12)

**Figure 7.**
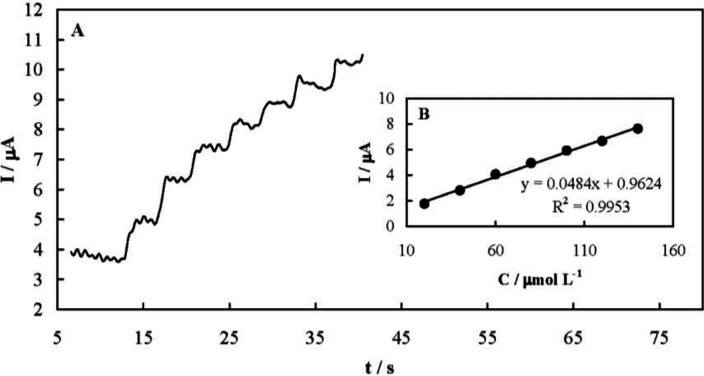
(A) Typical amperometric response of MCPE recorded upon successive famotidine increments; (B) dependency of the amperometric response of MCPE on the successive addition of famotidine

Amperometry was also employed for quantitative analysis of famotidine. Typical amperometric signals recorded upon successive famotidine increments are presented in Figure 7A. The solution was stirred after each injection and during the amperometric signals recording. The current responses were quite fast and dependent on the drug concentration as shown in Figure 7B. The analytical parameters of the amperometric method are presented in Table 1. In addition, Table 2 compares the determined analytical parameters of the developed amperometric method in this study with other reported techniques. The developed amperometric method was more simple, faster, and low-cost compared to the others.

**Figure 8:**
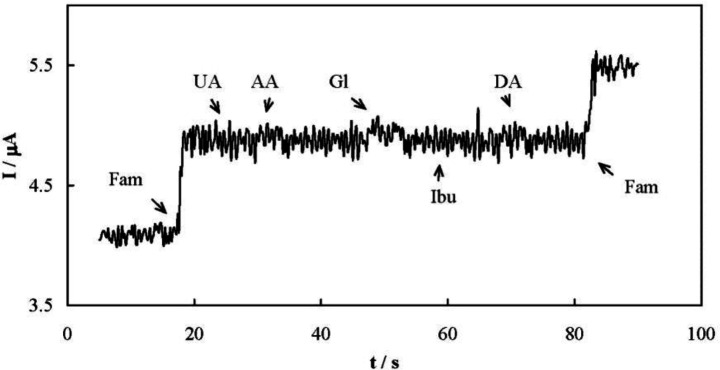
Amperometric response of MCPE covered with a layer of Nafion membrane in the presence of various biological compounds

Selectivity of the amperometric method for the determination of famotidine was evaluated. One of the most important interfering species during the famotidine determination is ibuprofen, since famotidine and ibuprofen are occasionally co-formulated in a mixture. The interfering effects of uric acid, ascorbic acid, glucose, and dopamine were also checked. Ibuprofen does not possess any oxidizable functional group and therefore is not electroreactive. On the other hand, all of the interfering species are negatively charged in the supporting electrolyte. When a Nafion membrane was applied to the MCPE surface, amperometric signals did not show any chemical interference in the presence of all these compounds due to the repulsion between these anionic compounds and the Nafion membrane (Figure 8).

The repeatability and reproducibility of the amperometric method was determined by repetitive measuring of the electrode response. Three concentrations of famotidine were analyzed by three independent measurements over one day (intra-day assay) and over three days (inter-day assay). Relative standard deviation (RSD) values of smaller than 5% were obtained for both the assays. In addition, a 20 μmol L^-1^ famotidine solution was analyzed three times using the same MCPE and a RSD value of 3.85% was obtained. Similar determinations were performed using three fabricated MCPE electrodes, resulting in a RSD value of 7.15%. These results suggest that the developed amperometric method exhibited desirable repeatability and reproducibility.

Application of the amperometry method for the determination of the drug in sample dosage forms was evaluated by analyzing the famotidine tablets. The amount of the drug in the tablets was determined by the amperometric method and the obtained results are presented in Table 3. Good agreement was observed for the drug analyzed using the presented method with the nominal values.

**Table 1:** The determined parameters for the calibration curve of famotidine and accuracy and precision using MCPE

Linear range/µmol L^-1^	20-140
Sensitivity (Slope)/mA L mol^-1^	48.4
Intercept/µA	0.96
R^2^	0.9953
Standard error of slope (*P* = 0.005)	1.5
Standard error of intercept (*P* = 0.005)	0.13
Detection limit/µmol L^-1^	5.91
Quantitation limit/µmol L^-1^	19.7
RSD^a^%	5.21

^ a^RSD is for 80 µmol L^-1^.

**Table 2:** A comparison between different methods of famotidine determination

**Method**	**Electrode**	**Linear range**	**Detection limit**	**Matrix**	**Reference**
HPLC	-	10-100 µg mL^-1^	10 µg mL^-1^	Tablet	(35)
Spectrophotometry	-	2.5-25 µg mL^-1^	0.62 µg mL^-1^	Tablet	(31)
SW AdSV	CG-MDE	0.17-20.2 µg mL^-1^	0.02 µg mL^-1^	Pure drug form	(40)
LS AdSV	CG-MDE	0.34-13.5 µg mL^-1^	0.06 µg mL^-1^	Pure drug form	(40)
HPLC	-	0.631-252 ng mL^-1^	0.252 ng mL^-1^	Plasma^a^	(37)
HPLC	-	0.631-252 ng mL^-1^	0.252 ng mL^-1^	Plasma^b^	(37)
HPLC	-	0.075-30.0 µg mL^-1^	0.015 µg mL^-1^	Urine^c^	(37)
Spectrophotometry	-	2-61 μg mL^-1^	0.28 μg mL^-1^	Pure drug form	(32)
Gas chromatography	-	80-700 ng mL^-1^	17 ng mL^-1^	Pure drug form	(38)
HPLC with UV detection	-	5-25 μg mL^-1^	0.134 μg mL^-1^	Pure drug form	(36)
Spectrofluorimetry	-	10-100 ng mL^-1d^	0.79 ng mL^-1e^	Pure drug form	(33)
Spectrofluorimetry	-	5-70 ng mL^-^^1f^	0.74 ng mL^-1g^	Pure drug form	(33)
Spectrofluorimetry	-	5-60 ng mL^-1h^	1.71 ng mL^-1i^	Pure drug form	(33)
CZE	-	1.5-48 μg mL^-1^	0.09 μg mL^-1^	Tablet	(39)
Potentiometry	ISME	3.37-337.5 mg mL^-1^	-	Tablet	(34)
Amperometry	MCPE	6.75-47.2 mg mL^-1^	1.99 mg mL^-1^	Tablet	This study

**Table 3:** Determination of famotidine in tablets

**Tablet**	**Amount labeled/mg**	**Amount found/mg**	**Bias%**
A	20	20.8	+4.0
B	20	19.4	-3.0
C	20	19.1	-4.5

## Conclusion

An electrochemical sensor based on flower-like nickel hydroxide nanosheets was designed and fabricated for famotidine. The developed sensor was employed to study the kinetics of the electrocatalytic oxidation of the drug using a variety of electrochemical techniques. Voltammetric studies showed a large anodic current at the oxidation potential of low-valence nickel hydroxide species in further support of the mediated electrooxidation. An electrochemical oxidation mechanism was proposed based on the oxidation of low-valance nickel species followed by the oxidation of famotidine on the electrode surface. In addition, the kinetic parameters of the drug including charge-transfer coefficient, catalytic reaction rate constant and coefficient of diffusion for oxidation were determined. Moreover, an amperometric procedure was successfully applied for the quantification of famotidine in pharmaceutical formulations. Comparison of the analytical parameters obtained for the fabricated sensor revealed the enhanced sensitivity and selectivity of the developed electrochemical analysis in this study.

## Supplementary Materials

Supplement
