# Supplementary material for: Synthesis of Flower-like Nickel Hydroxide Nanosheets and Application in Electrochemical Determination of Famotidine
Source: Iran J Pharm Res. 2020 Winter;19(1):120–37. doi: 10.22037/ijpr.2019.14257.12245 (PMC7462516; doi:10.22037/ijpr.2019.14257.12245)
Supplement: Supplement [file ijpr-19-120-s001.pdf]

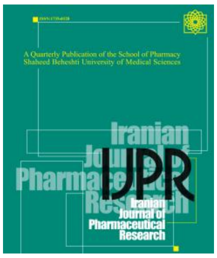

Supplementary Materials for

Synthesis of Flower-like Nickel Hydroxide Nanosheets and Application in Electrochemical Determination of Famotidine

Rezvan Dehdari Vais, Hossein Yadegari and Hossein Heli*

*To whom correspondence should be addressed. E-mail: hheli7@yahoo.com

Volume 19, Issue 1 (Winter 2020)

This PDF file includes:

Figure S1

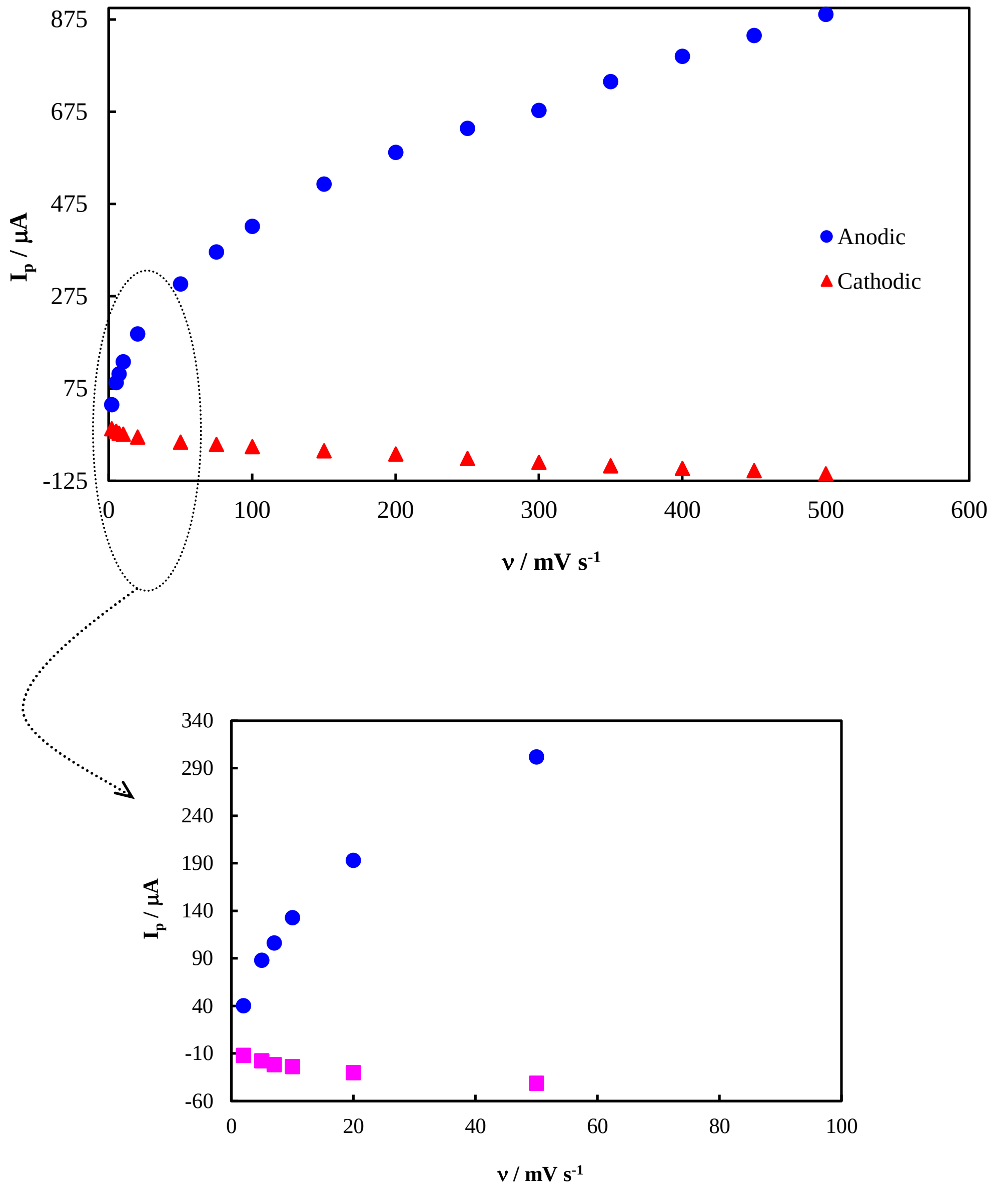

Figure S1. Dependencies of the peak currents on the potential sweep rate. Upper part: main panel; lower part: enlargement of the main panel for small values of the potential sweep rate.
